# A genome-wide association study identified loci for yield component traits in sugarcane (*Saccharum* spp.)

**DOI:** 10.1371/journal.pone.0219843

**Published:** 2019-07-18

**Authors:** Fernanda Zatti Barreto, João Ricardo Bachega Feijó Rosa, Thiago Willian Almeida Balsalobre, Maria Marta Pastina, Renato Rodrigues Silva, Hermann Paulo Hoffmann, Anete Pereira de Souza, Antonio Augusto Franco Garcia, Monalisa Sampaio Carneiro

**Affiliations:** 1 Departamento de Biotecnologia e Produção Vegetal e Animal, Centro de Ciências Agrárias, Universidade Federal de São Carlos, Araras, São Paulo, Brasil; 2 Departamento de Genética, Escola Superior de Agricultura Luiz de Queiroz, Universidade de São Paulo, Piracicaba, São Paulo, Brasil; 3 Centro de Pesquisa e Desenvolvimento de Cultivares de Soja, Setor de Pesquisa e Desenvolvimento, FTS Sementes S.A., Ponta Grossa, Paraná, Brasil; 4 Centro de Pesquisa e Desenvolvimento, Embrapa Milho e Sorgo, Sete Lagoas, Minas Gerais, Brasil; 5 Instituto de Matemática e Estatística, Campus Samambaia, Universidade Federal de Goiás, Goiânia, Goiás, Brasil; 6 Departamento de Biologia Vegetal, Instituto de Biologia, Universidade Estadual de Campinas, Campinas, São Paulo, Brasil; 7 Centro de Biologia Molecular e Engenharia Genética, Universidade Estadual de Campinas, Campinas, São Paulo, Brasil; USDA-ARS Southern Regional Research Center, UNITED STATES

## Abstract

Sugarcane (*Saccharum* spp.) has a complex genome with variable ploidy and frequent aneuploidy, which hampers the understanding of phenotype and genotype relations. Despite this complexity, genome-wide association studies (GWAS) may be used to identify favorable alleles for target traits in core collections and then assist breeders in better managing crosses and selecting superior genotypes in breeding populations. Therefore, in the present study, we used a diversity panel of sugarcane, called the Brazilian Panel of Sugarcane Genotypes (BPSG), with the following objectives: (i) estimate, through a mixed model, the adjusted means and genetic parameters of the five yield traits evaluated over two harvest years; (ii) detect population structure, linkage disequilibrium (LD) and genetic diversity using simple sequence repeat (SSR) markers; (iii) perform GWAS analysis to identify marker-trait associations (MTAs); and iv) annotate the sequences giving rise to SSR markers that had fragments associated with target traits to search for putative candidate genes. The phenotypic data analysis showed that the broad-sense heritability values were above 0.48 and 0.49 for the first and second harvests, respectively. The set of 100 SSR markers produced 1,483 fragments, of which 99.5% were polymorphic. These SSR fragments were useful to estimate the most likely number of subpopulations, found to be four, and the LD in BPSG, which was stronger in the first 15 cM and present to a large extension (65 cM). Genetic diversity analysis showed that, in general, the clustering of accessions within the subpopulations was in accordance with the pedigree information. GWAS performed through a multilocus mixed model revealed 23 MTAs, six, three, seven, four and three for soluble solid content, stalk height, stalk number, stalk weight and cane yield traits, respectively. These MTAs may be validated in other populations to support sugarcane breeding programs with introgression of favorable alleles and marker-assisted selection.

## Introduction

Sugarcane (*Saccharum* spp.) is an important industrial crop and a vital component for food and energy security, providing sucrose, bioethanol and bioelectricity [1,2]. Sugarcane is cultivated in mainly tropical and subtropical areas and has a very high photosynthetic efficiency and a complex genome due to its variable ploidy levels, frequent aneuploidy, and large genome size of approximately 10 gigabases (Gb) [3–8]. Modern sugarcane cultivars have chromosome numbers ranging from 100 to 130, are vegetatively propagated, and result from the selection of populations derived from outcrossing heterozygous parents [8–10]. Brazil is the world’s largest sugarcane producer, and its productivity increased 66% in tons of sugarcane per hectare from 1975 to 2010, partially due to the growing area expansion and improvements in agricultural practices [2,10,11].

Sugarcane breeding programs concentrate efforts to release cultivars adapted to different environments that have high yields in terms of biomass production and sucrose content as well as resistance to diseases. However, the breeding process is expensive and requires approximately 15 years of experimentation and selection to obtain one or a few cultivars. Briefly, every year, crosses between accessions generate hundreds of thousands of F1 progenies, and the individuals reaching the final stages of selection are commonly evaluated over several harvests in multienvironment trials (METs) to identify those with the potential to become new cultivars [10–13]. Even with the adoption of better agricultural practices and selection strategies in the early stages of breeding programs, which attempt to measure and isolate the environmental effects of genetic factors [13–16], the genetic gains to quantitative traits have declined in recent years for sugarcane and other crops [17–19].

Clearly, there is a need to complement the classical breeding of sugarcane with other tools, such as molecular approaches, which have been applied for other crops [20–22]. Quantitative trait locus (QTL) mapping and genome-wide association studies (GWAS) are strategies to understand the genetic architecture of complex traits and include a first step of marker-assisted selection (MAS) [4,6,19,22]. To employ these strategies in outcrossing heterozygous species, such as sugarcane, we need to consider that, for each segregating locus, different numbers of segregating alleles may exist, and the single-dose markers currently available for mapping studies show only some of the genetic information [8,23]. This limitation is more evident in the traditional QTL mapping approach, which may identify genomic regions with low resolution, usually due to the smaller amount of available markers and also limited to the genetic composition of the biparental population under study. Nevertheless, attempts to associate phenotype and genotype and the development of new data analysis strategies have been significantly advanced [23–27].

On the other hand, GWAS has been widely used to identify marker-trait associations (MTAs) in genetically diverse populations of plants [20,21,28–32]. GWAS is based on linkage disequilibrium (LD) due to physical linkage, which is reportedly extensive in sugarcane [33–36]. This LD value is assigned to a recent breeding history, characterized by a strong foundation bottleneck followed by a small number of intercrossing cycles, which significantly reduces the frequency of recombination events. The high extent of LD in sugarcane indicates that a high density of markers may not be critical for performing GWAS [36–38] and that single-dose markers might be appropriate for this purpose; indeed, mapping models for loci with high allelic dosages are under development [26]. Although high-throughput marker systems are available, mainly for single nucleotide polymorphism (SNP) genotyping, the lack of appropriate methods for analyzing complex species such as sugarcane hinders the applicability of new molecular breeding tools [23,26,27]. In this context, single-dose markers, such as simple sequence repeats (SSRs) and target region amplification polymorphisms (TRAPs) could be used to characterize genome variation, investigate population structure and genetic diversity and thus enable GWAS [37,39–41]. In addition, despite the potential for using LD-based association studies to identify MTAs, a few studies on yield-related traits in sugarcane have been published [18,35–42].

For the latter, several algorithms and software have been developed to improve statistical power, increase computational efficiency, and reduce spurious associations in the GWAS approach [43]. Among GWAS algorithms, FarmCPU [44], which uses a multilocus linear mixed model (MLMM), is considered an efficient alternative to control for spurious associations [45–47]. Indeed, combinations of various methods for multilocus GWAS have also been used to identify causal associations and control the false positive rate [43,47,48].

In the current assignment, our objectives were to (i) estimate, through a mixed model, the adjusted means and genetic parameters of the five yield traits evaluated over two harvest years in a diversity panel composed of ancestral and modern sugarcane accessions; (ii) detect population structure, LD and genetic diversity using SSR markers; (iii) perform GWAS analysis to identify MTAs; and iv) annotate the sequences giving rise SSR markers that had fragments associated with target traits to search for putative candidate genes.

## Materials and methods

### Plant material and phenotypic traits

In this study, 134 accessions (S1 Table) of the Brazilian Panel of Sugarcane Genotypes (BPSG) were used. BPSG is a mini core collection from the germplasm bank of RIDESA (Inter-University Network for the Development of Sugarcane Industry), and the accessions were chosen according to the following criteria: i) relevant Brazilian cultivars, ii) main parents for Brazilian breeding programs; iii) cultivars from countries that grow sugarcane; iv) cultivars used as parents in mapping programs [25,49]; and v) representatives of the *Saccharum* species complex. The BPSG accessions represent an important genetic background in Brazilians breeding programs.

The 134 accessions of BPSG were planted in a field experiment performed in 2013 at the Agricultural Science Center of the Federal University of São Carlos (UFSCar) in Araras City, São Paulo State, Brazil. Araras is located at 22°21’25”S, 47°23’3”W at an altitude of 611 m; the experimental area soil is Typic Eutroferric Red Latosol. The experimental design consisted of a randomized complete block, which was fully replicated four times. The plots consisted of two rows 3 m long and spaced 1.5 m apart. Each plot was composed of 12 presprouted seedlings at the planting of the experiment in 2013. The experimental plants were harvested when they were approximately 18 months of age during the plant cane and first ratoon. The BPSG was evaluated for five yield components: soluble solid content (BRIX, in °Brix), stalk height (SH, in m), stalk number (SN), stalk weight (SW, in kg), and cane yield (TCH, in t ha^–1^). Phenotypic yield trait data were collected according to Balsalobre et al. [12]. Briefly, a 10-stalk sample per plot was taken for analysis of the BRIX and SH. The weight of the 10 stalks was added to the total weight of the plot (SW) to estimate the TCH, which was calculated as the product between the SW of a linear meter and the amount of linear meters in one ha (6667 linear meters compose one ha with a spacing of 1.5 m). The SN was estimated by directly counting the stalks in each plot.

### Statistical analysis of phenotypic data

A multiharvest mixed model produced the joint adjusted means. The analysis was conducted for each trait using GenStat 19th edition [50] based on restricted maximum likelihood (REML) and the following linear mixed model:

$$y_{imkuv}=\mu +h_{m}+b_{km}+g_{imk}+r_{umk}+c_{vmk}+e_{imkuv}$$

where *y*_*imkuv*_ is the phenotype of the *i*^th^ accession, evaluated in the *m*^th^ harvest, located in the *u*^th^ row and the *v*^th^ column inside the *k*^th^ replication; μ is the overall mean; *h*_*m*_ is the fixed effect of the *m*^th^ harvest (*M* = 1,…,*M*;*M* = 2); *b*_*km*_ is the fixed effect of the *k*^th^ replication (*k* = 1,…,*K*;*K* = 4) at the *m*^th^ harvest; *g*_*imk*_ is the random effect of the *i*^th^ accession (*i* = 1,…,*I*,*I* = 134) at the *m*^th^ harvest evaluated in the *k*^th^ replication; *r*_*umk*_ and *c*_*vmk*_ are the random effects of the *u*^th^ row and *v*^th^ column, both evaluated at the *m*^th^ harvest and *k*^th^ replication; and *e*_*imkuv*_ is the random residual error. In addition, for the SN, SW, and TCH traits, the number of clumps per plot was included in the mixed model as a fixed covariate. Aiming to model the accession effects, the genetic variance–covariance (VCOV) matrix **G** = **G**_*M*_ ⊗ **I**_*Ig*_, i.e., **g**~N(**0**,**G**) was considered, where *M* is the number of harvests, and ⊗ represents the Kronecker product of both the genetic **G**_*M*_ and identity **I**_*Ig*_ matrices with the respective dimensions of 2 *x* 2 and 1 *x* 134. For the **G**_*M*_ matrix, four structures (identity, ID; diagonal, DIAG; first order autoregressive homogenous, AR1; and first order autoregressive heterogeneous, AR1(het)) were examined and compared via Akaike [51] (AIC; [51]) and Bayesian (BIC; [52]) information criteria [53]. For the residue, a structure of variance heterogeneity was assumed for the different harvests. For each trait, the fixed effects were tested using the Wald statistics test and were retained in the model if statistically significant (*P* < 0.05). After the **G**_*M*_ matrix structure selection, the adjusted means for accessions and genetic parameters for each evaluated trait were obtained. The phenotypic ${(\hat{\sigma }}_{P}^{2})$ and genotypic (${\hat{\sigma }}_{g}^{2}$) variances were used for calculating heritability in the broad sense on an individual-plant basis (${\hat{H}}^{2}={\hat{\sigma }}_{g}^{2}/{\hat{\sigma }}_{P}^{2}$). The ${\hat{\sigma }}_{P}^{2}$ value was determined from ${\hat{\sigma }}_{P}^{2}={\hat{\sigma }}_{g}^{2}+{\hat{\sigma }}_{e}^{2}+{\hat{\sigma }}_{r}^{2}+{\hat{\sigma }}_{c}^{2}$, where ${\hat{\sigma }}_{e}^{2}$ was the residual variance, ${\hat{\sigma }}_{r}^{2}$ was the variance for row effects and ${\hat{\sigma }}_{c}^{2}$ was the variance for column effects [54].

### DNA extraction, molecular markers and genotyping

Approximately 3.0 g of tissue from the leaf primordia of each accession was collected, and the genomic DNA was extracted according to methods described by Aljanabi et al. [55]. The SSR markers were amplified based on the procedures described by Oliveira et al. [56], and the amplified fragments were visualized as described by Creste et al. [57]. A total of 100 SSR primers were used, of which 86 were from expressed sequences (EST-SSR) [58,59] and 14 were of genomic origin [60]. These markers were selected because they met one or more of the following criteria: i) high polymorphic information content (PIC); ii) high discrimination power (DP); and iii) present in previously published sugarcane genetic maps.

Due to the polyploid and complex nature of sugarcane, the amplified SSR fragments, which cannot depict ploidy levels and allele dosages, were evaluated as dominant markers [61,62], i.e., the presence of fragments suggested that an allele for a given locus was present in at least one of the chromosomes that comprised a homologous group, while the absence of fragments suggested that this same allele was not present in any chromosome. Thus, the fragments were classified as binary, i.e., (1) indicated a fragment was present, and (0) indicated a fragment was absent. When amplification failed, NA (nonamplified) was used to indicate missing data. The polyacrylamide gels were manually evaluated with the support of a light box, and a binary matrix formed by the combination of the detected fragments with the analyzed accessions was constructed.

### Population structure and genetic diversity

Population structure was analyzed by a discriminant analysis of principal components (DAPC) [63] using SSR data in the adegenet package [64], which is available in R software [65], as described by Jombart and Collins [66] and Deperi et al. [67]. Briefly, the find.clusters function was used to detect the number of clusters in the BPSG. This function uses K-means clustering, which decomposes the total variance of a variable into between-group and within-group components. The best number of subpopulations has the lowest associated BIC. A cross validation function (xval.Dapc) and optimal α-score function (optim.a.score) were used to confirm the correct number of principal components (PCs) to be retained. The optimal number of PCs to retain is associated with the lowest root mean square error and with the highest optimized α-score. The subpopulations indicated by DAPC were plotted in a scatterplot considering the first and second linear discriminants. Additionally, a genetic dissimilarity matrix was calculated via a simple matching (SM) method using Darwin software [68] based on the SSR information. Then, the resulting matrix was plotted as a phylogram using the neighbor-joining (NJ) algorithm [69]. In addition, bootstrap analysis was performed as described by Efron [70] and Efron and Tibshirani [71] to verify whether the number of fragments evaluated was sufficient to distinguish the accessions. The coefficients of variation are graphically shown as boxplots for each sampling with different numbers of fragments.

### Kinship matrix

The kinship coefficient was calculated between pairs of accessions using the kinship2 package [72] in R, considering the accessions of all generations and assigning the value 0 when the parents were unknown. Based on the estimated kinship coefficients, a kinship matrix (*K*) was generated.

### Linkage disequilibrium analysis

Marker data were used to assess the level of LD in the BPSG as described by Raboin et al. [35]. Briefly, Fisher’s exact probability was used to test for associations between SSR fragments that were common to both the association mapping population and the SP80-180 and SP80-4966 integrated genetic map [56]. For each pair of markers, a contingency table (presence versus absence) was established, and the Fisher probability was computed using the exact2x2 package in R software [73]. To control for error due to multiple testing, we used the false discovery rate (FDR) procedure [74] with an initial threshold of 5%. A Bonferroni-corrected threshold was also verified. The Fisher (−*LogP*) logarithmic probabilities of the associations between only linked fragments were plotted with the respective genetic distances [75] in centimorgans (cM).

### GWAS analysis

GWAS analysis was conducted using both the Genomic Association and Prediction Integrated Tool (GAPIT, [76]) and FarmCPU [44] methods in R software. To carry out GWAS analyses using the SSR data obtained in the BSPG, the fragments were reclassified, with (2) indicating the presence of a fragment and (0) indicating the absence of a fragment. The retained PC obtained in DAPC analysis was used as a covariate in the FarmCPU procedure, while the kinship matrix and retained PC were used in the GAPIT analysis. To control for type I errors due to multiple testing, the adjusted *p*-value less than 1% following an FDR controlling procedure [77] and Bonferroni-corrected threshold with 1% were used to declare significant MTAs by GAPIT and FarmCPU, respectively. To determine which of the tested methods best fit the data, we plotted the quantile-quantile (QQ) plot, i.e., the QQ negative log10-transformed observed *p*-values obtained for each MTA, against their expected distribution under the null hypothesis of no genetic association. For significant MTAs detected by FarmCPU, the phenotypic variance explained for each SSR fragment was estimated one at a time using a linear model with the lm function in R software.

### Sequence annotation

Functional annotation of the loci associated with traits was performed using the available sequences that gave rise to the SSR marker. These sequences were annotated using i) the nonredundant NCBI database with *e*-values ≤ 1 × 10^−3^ through BLASTX and ii) the Phytozome website [78], which was used to align the data against the *Viridiplantae* protein databases.

## Results

### Phenotypic data

The VCOV models selected for the **G**_*M*_ matrix were based on AIC and BIC criteria. AR1(het) had the lowest AIC and BIC values, which indicated that it was the best model for all evaluated traits (BRIX, SH, SN, SW and TCH) (S2 Table). This result supports heterogeneous genetic variances between harvests and correlations between successive harvests and provides a systematic explanation of the existing temporal dependence. The ranges, adjusted means and estimates of the components of variance, coefficients of variation, and broad-sense heritability on an individual-plant basis for the five traits evaluated for the BPSG over the two harvest years (plant cane and first ratoon) are summarized in Table 1. The TCH trait had the highest variation, i.e., the accession RB925268 (295.60 t ha^-1^) was 7.6 times greater than the accession POJ2878 (38.90 t ha^-1^). The SN trait also showed high variation, i.e., the accession IN84-58 (290.64 stalks) was 7.03 times greater than the accession POJ2878 (41.34 stalks). On the other hand, the BRIX trait had a relatively low variation, i.e., the accession TUC71-7 (22.55°Brix) was 1.48 times greater than the accession IN84-58 (15.14°Brix).

**Table 1:** Ranges, adjusted means, estimates of components of genetic variance (${\hat{\sigma }}_{G}^{2}$) and phenotypic variance (${\hat{\sigma }}_{P}^{2}$), coefficients of genetic variation (CV_G_) and phenotypic variation (CV_R_), and broad-sense heritability on an individual-plant basis (${\hat{H}}^{2}$) for BRIX, SH, SN, SW and TCH for the BPSG over two harvest years (plant cane (1) and first ratoon (2)).

Trait	Range	Adjusted means	${\hat{\sigma }}_{G(1)}^{2}$	${\hat{\sigma }}_{G(2)}^{2}$	${\hat{\sigma }}_{P(1)}^{2}$	${\hat{\sigma }}_{P(2)}^{2}$	*CV*_*G*(1)_	*CV*_*G*(2)_	*CV*_*P*(1)_	*CV*_*P*(2)_	${{\hat{H}}^{2}}_{\left(1\right)}$	${{\hat{H}}^{2}}_{\left(2\right)}$
BRIX (°Brix)	15.14–22.55	18.65	1.45	0.96	1.09	0.72	0.06	0.05	0.05	0.04	0.57	0.57
SH (m)	1.51–3.08	2.64	0.08	0.08	0.06	0.06	0.11	0.11	0.09	0.09	0.57	0.57
SN	41.34–290.64	81.77	482.60	1197.00	232.10	645.40	0.27	0.42	0.18	0.31	0.67	0.65
SW (kg)	47.80–251.80	152.30	2182.00	1602.00	1576.00	1267.00	0.31	0.26	0.26	0.23	0.58	0.56
TCH (t ha^-1^)	38.90–295.60	181.40	2740.00	2127.00	2891.00	2192.00	0.29	0.25	0.30	0.26	0.48	0.49

Estimates for ${\hat{H}}^{2}$ ranged from 0.48 (TCH) to 0.67 (SN) and from 0.49 (TCH) to 0.65 (SN) in the first and second harvests, respectively. For genetic (${\hat{\sigma }}_{G}^{2}$) and phenotypic (${\hat{\sigma }}_{P}^{2}$) variances, higher and lower values were observed for the TCH and SH traits, respectively. The lowest coefficients of genetic (CV_G_) and phenotypic (CV_P_) variations were for the BRIX trait, while the higher values for CV_G_ and CV_P_ were for SN, SW and TCH.

**Fig 1:**
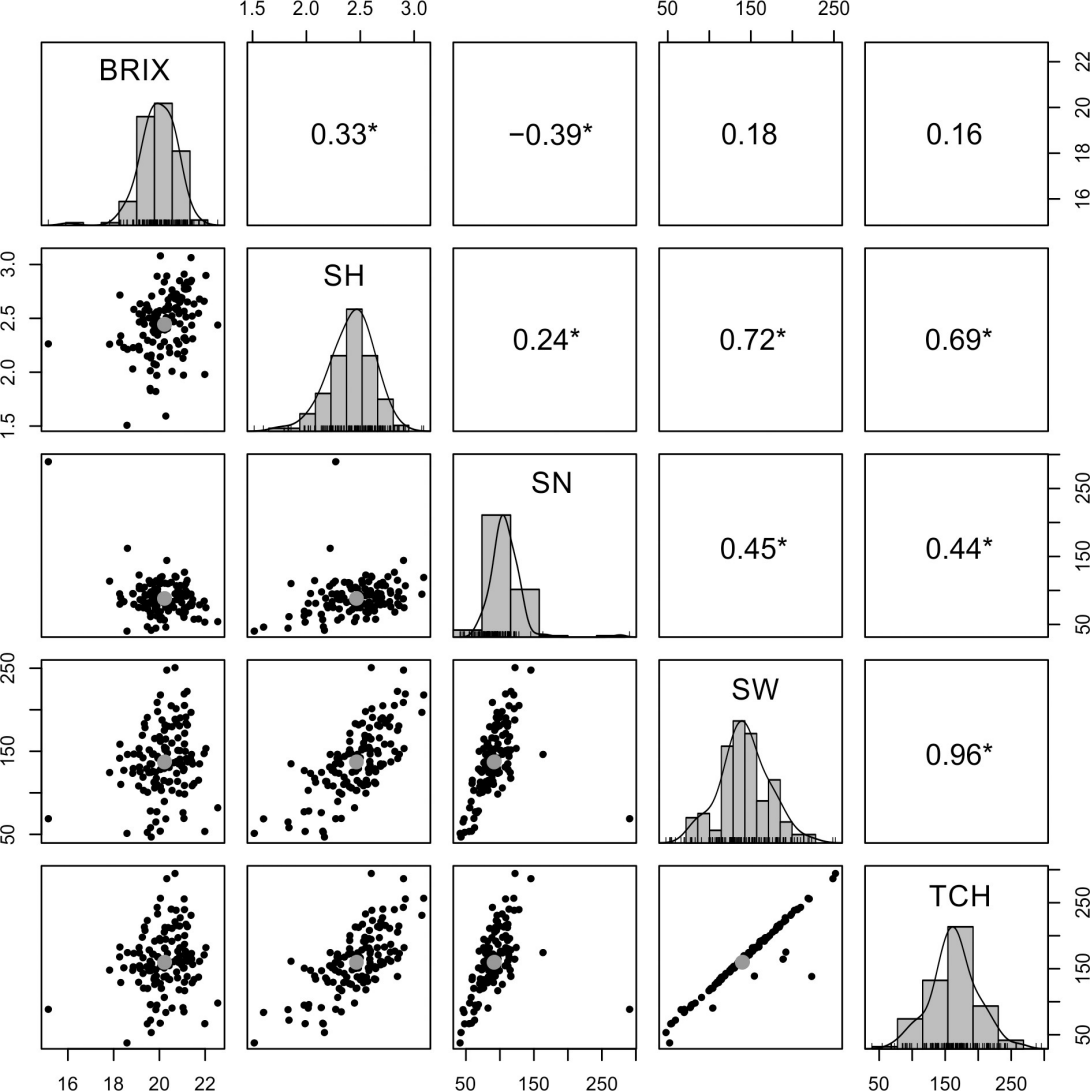
Genotypic correlation between yield traits evaluated in the BPSG. For each trait, the histograms of the adjusted means (diagonal), scatterplots (below diagonal), and values of the genotypic correlation (above diagonal) between pairs of traits are shown. *Significant at the 5% global level (*P* < 0.05).

Pairwise genotypic correlations among the five evaluated traits, considering both harvests (plant cane and first ratoon), are shown in Fig 1. In total, eight significant genotypic correlations (*P* < 0.05) were observed between the evaluated traits in the BPSG. According to the degree of correlation between traits, correlations were grouped into low (≤0.35), moderate (0.36–0.70) and strong (≥0.71) categories [12]. Thus, four interactions were classified as low (BRIX–SH, BRIX–SW, BRIX–TCH and SH–SN), four interactions were classified as moderate (BRIX–SN, SN–SW, SN–TCH and SH–TCH), and two interactions were classified as strong (SH–SW and SW–TCH). The correlation of BRIX–SN was negative.

### Polymorphisms of SSR markers

The use of 100 SSR markers generated 1483 fragments, 1476 of which were polymorphic (99.5%), in the 134 accessions of the BPSG. Considering all polymorphic fragments, 484 (32.8%) were produced by SSR dinucleotides, 689 (46.7%) were produced by SSR trinucleotides, and 303 (20.5%) were produced by SSR tetranucleotides. The number of fragments ranged from four (ESTC52 and ESTC55) to 36 (ESTA31), with an average of 14.83 fragments per SSR. Species-specific fragments were observed for the ancestral accessions Badila (*S*. *officinarum*) at ESTB45 and SMC319; Ganda Cheni (*S*. *barberi*) at ESTB45, ESTB118, ESTA51, and ESTC17; and especially IN84-58 (*S*. *spontaneum*) at CIR23, ESTA26, ESTA61, CIR55, ESTB69, ESTA33, ESTB94, ESTA63, CIR18, ESTB63, CIR36, ESTB45, ESTA16, ESTC55, ESTA48, SMC222 and CIR25.

### Population structure and genetic diversity

**Fig 2:**
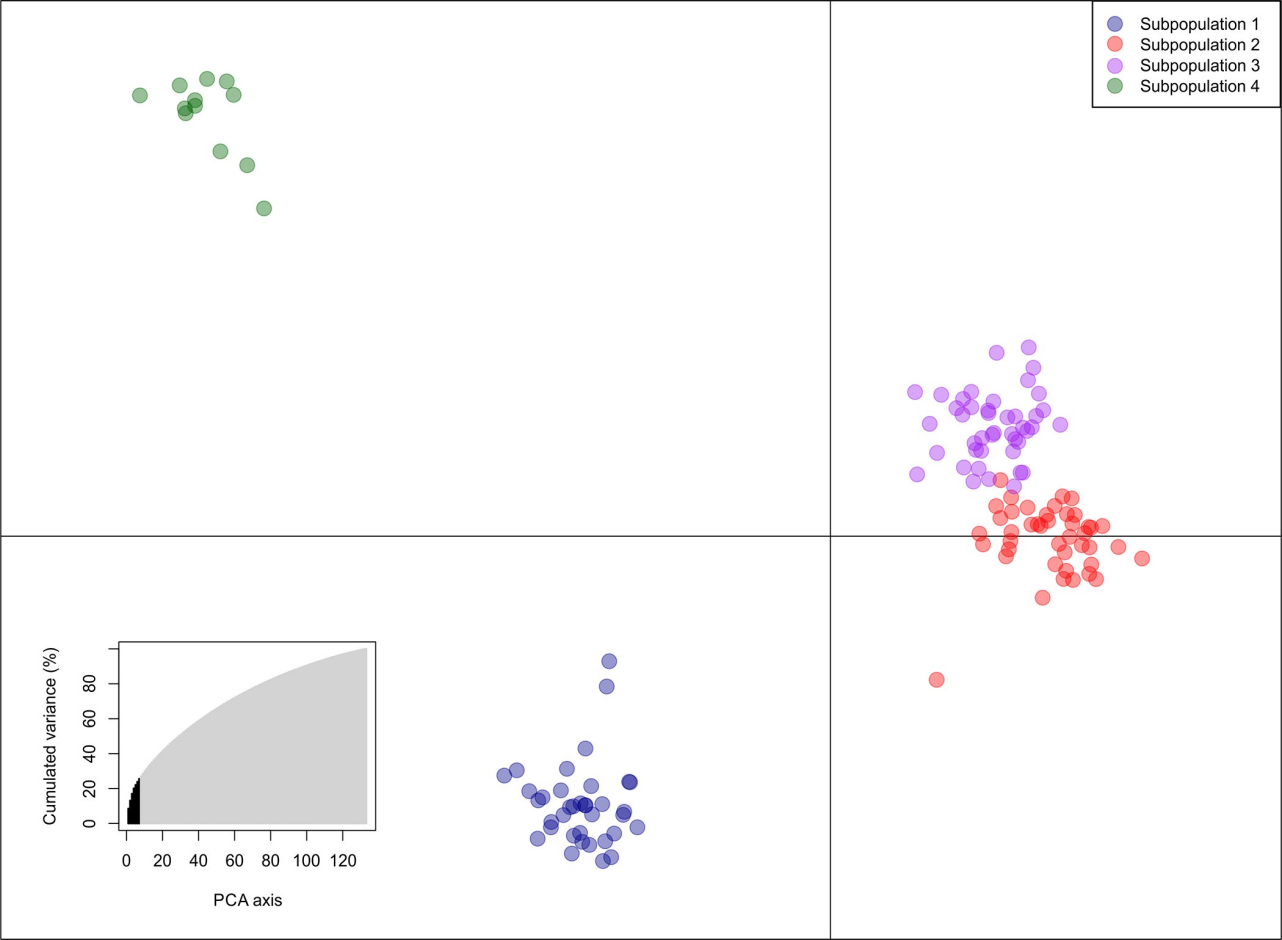
DAPC for the BPSG. The axes represent the first two linear discriminants (LD). The dots represent accessions grouped in subpopulations, each with a different color. The cumulative variance values, in percentages, of the PCs are shown in the lower left corner of the figure; the eigenvalues of the seven first PCs retained by PCA are in black.

Four subpopulations were detected according to the lowest BIC value derived by the find.clusters function (S1 Fig). DAPC analysis was performed using the detected number of subpopulations (Fig 2). Seven first PCs (25.5% of variance conserved) from principal component analysis (PCA) (S2 and S3 Figs) and three discriminant eigenvalues were retained. All accessions were classified in each subpopulation with a membership coefficient equal to 1, suggesting that there were no admixtures and that the BPSG was structured (S4 Fig). A total of 42 fragments with the largest contribution to subpopulation identification were detected, with 24 fragments assigned to linear discriminant 1 and 18 fragments assigned to linear discriminant 2 (S3 Table and S5 Fig).

**Fig 3:**
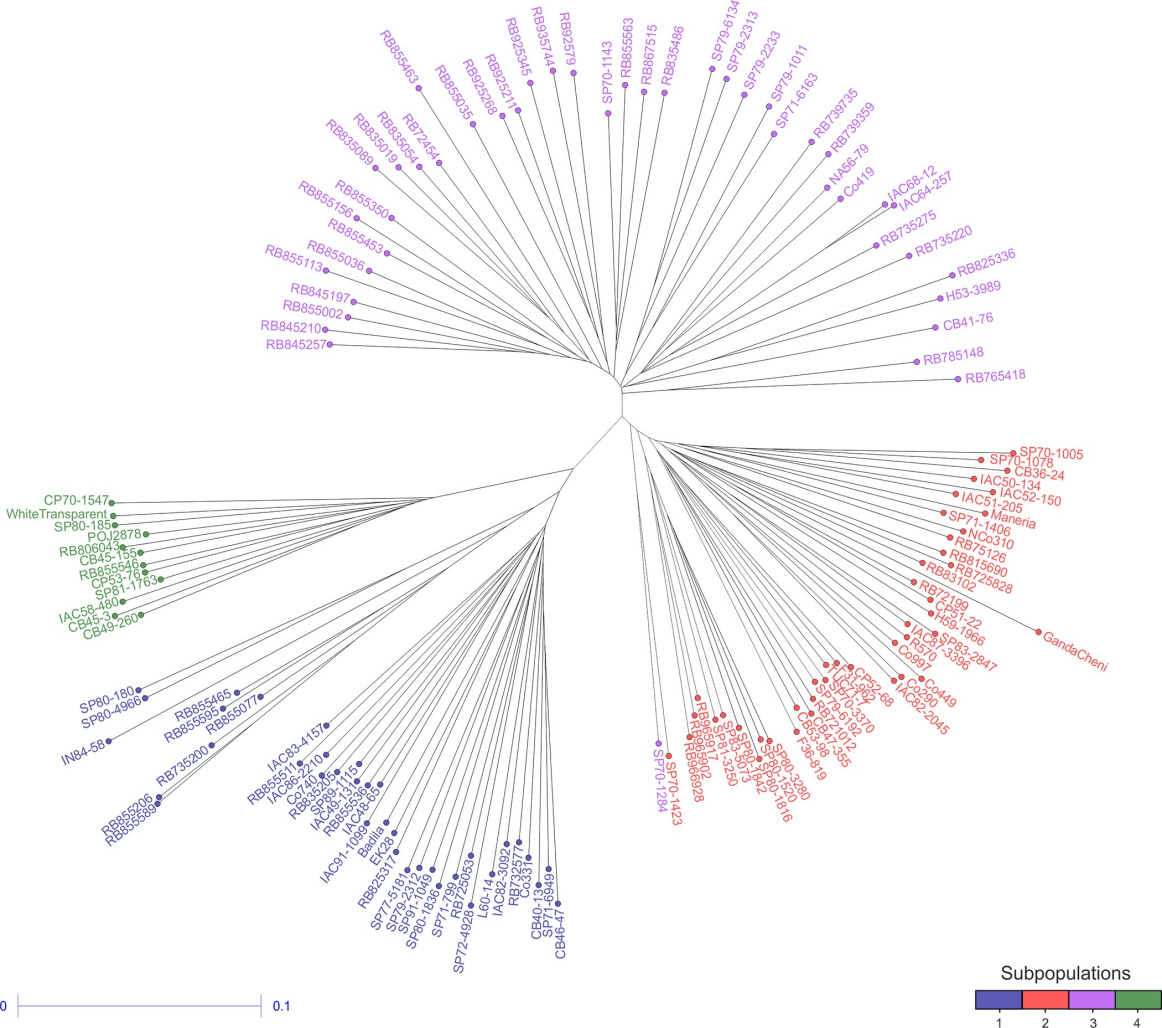
Neighbor-joining (NJ) tree for the BPSG using the SM method. Accessions indicated with the same color belong to the same subpopulation according to DAPC.

The phylogram using the SM genetic distance among accessions also suggested the presence of four subpopulations. A total of 99.25% of the group assignments made by the DAPC analysis were also made by the phylogram (Fig 3). Only accession SP70-1284 was assigned to different groups by the NJ phylogram and DAPC methods. The genetic dissimilarity ranged from 0.06 (between accessions IAC68-12 and IAC64-257, in subpopulation 3) to 0.45 (between accessions SP70-1005 and RB855589, in subpopulations 2 and 1, respectively), with an average value of 0.31 (S6 Fig). Overall, the clusters inside subpopulations were in accordance with the pedigree information. This result was verified by full-sib accessions within the subpopulations, as was the case for the accessions RB845197, RB845210 and RB845257 in subpopulation 3, which originated from the crossing between cultivars RB72454 and SP70-1143, and for the cultivars SP80-1816, SP80-1842 and SP80-3280 in subpopulation 2, which originated from the crossing between the cultivars SP71-1088 and H57-5028. In addition, the ancestral accessions Maneria (*Saccharum sinense*) and Ganda Cheni (*S*. *barberi*) were placed in subpopulation 2, the ancestral accessions Badila (*S*. *officinarum*) and IN84-58 (*S*. *spontaneum*) were positioned in subpopulation 1, and the ancestral accession White Transparent (*S*. *officinarum*) was positioned in subpopulation 4.

### Linkage disequilibrium analysis

Fisher's (−*LogP*) logarithm probabilities were plotted against the distances, in centiMorgan (cM), between linked marker fragments in the same cosegregation group of the SP80-180 × SP80-4966 integrated genetic map (S7 Fig). This strategy corresponded to 60 of the 5151 associations between the 102 common SSR fragments being present in both the BPSG and the integrated genetic map. Although few significant associations reported LD (5 of the 60 associations), the population showed good evidence of LD decay in relation to genetic distance. The strongest LD appeared in the first 15 cM, mainly in the first 5 cM, and clear decay occurred over distances. In addition, LD was noted between fragments at 65 cM in the same cosegregation group, indicating preferential associations in larger extensions.

### GWAS analysis

**Fig 4:**
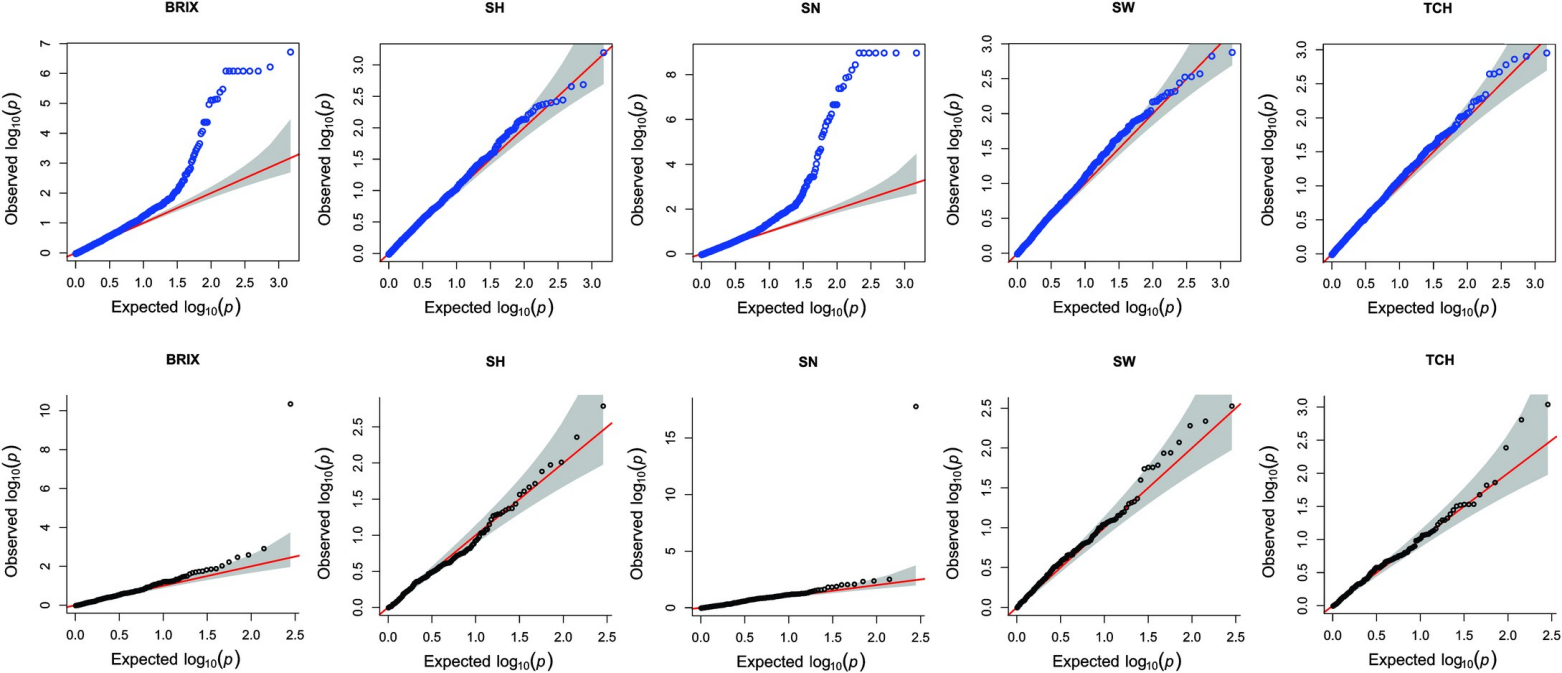
QQ plots using GAPIT (graphs with blue dots) and FarmCPU (graphs with black dots) software. The dotted lines show the 95% confidence intervals for the QQ plots under the null hypothesis of no association between the SSR fragment and the trait.

The QQ plots obtained with FarmCPU and GAPIT software for phenotypic traits are presented individually in Fig 4. The results show that FarmCPU compared to GAPIT better fit the data by reducing false positives, mainly for the BRIX and SN traits. Therefore, we considered the MTAs identified by FarmCPU to be more reliable than those identified by GAPIT and thus present the results of the former. For the BRIX, SH, SN, SW and TCH traits, 6, 3, 7, 4 and 3 MTAs were detected, respectively, with a Bonferroni-corrected threshold of 1% (Table 2). The SSR fragment ESTB61_15 was negatively and positively associated with BRIX and SN, respectively. ESTB61_15 is a species-specific fragment for *S*. *spontaneum* (IN84-58). The three SSR fragments associated with TCH were also associated with SW, and two of these fragments (CIR51_11 and SMC319_09) were in the group of marker fragments associated with SH. Although not in the same fragment as TCH, SW and SN, the genomic SSR marker SMC319 was also present among the SH MTAs and was therefore associated with four yield-related traits. Likewise, the genomic SSR marker CIR51 was associated with four yield-related traits, namely, BRIX, SN, SW and TCH.

**Table 2:** BRIX, SH, SN, SW and TCH MTAs, *p*-values, effect estimates and amounts of phenotypic variance explained (adjusted R-squared) when using the MLMM implemented in FarmCPU.

Trait	Code	SSR fragment	*p*-value	Effect	*R*^2^
BRIX	m93	ESTA61_07	0.009004	-0.22	0.03
BRIX	m101	ESTA61_15	4.29E-11	-2.79	0.20
BRIX	m131	CIR55_06	0.001163	0.24	0.02
BRIX	m139	CIR55_14	0.002446	-0.32	0.14
BRIX	m515	CIR51_04	0.005713	0.26	0.01
BRIX	m797	ESTB133_10	0.003254	0.27	0.01
SH	m745	SMC319_16	0.004327	-0.42	0.07
SH	m752	SMC248_08	0.001605	0.09	0.14
SH	m839	ESTC19_12	0.009647	-0.08	0.07
SN	m101	ESTA61_15	1.50E-06	104.29	0.43
SN	m138	CIR55_13	0.003936	21.06	0.02
SN	m522	CIR51_11	0.008232	5.37	0.04
SN	m650	ESTB111_05	0.008690	-11.21	0.02
SN	m664	ESTB111_19	0.002828	7.22	0.01
SN	m738	SMC319_09	0.007744	-5.59	0.03
SN	m921	ESTB130_16	0.004402	7.57	0.03
SW	m522	CIR51_11	0.004541	12.01	0.02
SW	m738	SMC319_09	0.002941	-12.98	0.03
SW	m937	ESTB130_32	0.008385	10.92	0.05
SW	m1070	SMC222_01	0.005182	-13.88	0.02
TCH	m522	CIR51_11	0.000901	15.94	0.03
TCH	m738	SMC319_09	0.001533	-15.78	0.03
TCH	m1070	SMC222_01	0.004051	-16.33	0.02

### Sequence annotation

The available sequences of the SSR markers significantly associated with the BRIX, SH, SN, SW and TCH traits were blasted against the nonredundant NCBI database using BLASTX and against the *Viridiplantae* protein database using Phytozome (Table 3). Sequence similarity was found for seven out of the ten SSR markers significantly associated with homologies for *Sorghum bicolor* (for the BRIX, SN and SW traits) and *Zea mays* (for the SH trait). A functional description of the sequences showed possible candidate genes for all traits except for TCH. Despite this result, the CIR51 marker, which was found near (approximately 5.3 kb) the *cytochrome P450* transcript region in *S*. *bicolor*, had fragments significantly associated with TCH in addition to BRIX, SN and SW. Overall, the homologies found for significant SSR markers associated with BRIX (ESTA61, ESTB133) suggest a role in the accumulation and trafficking of lipids and sucrose, while the homologies for significant SSR markers associated with SH (ESTC19), SN (ESTB111, ESTB130) and SW (ESTB130) were related to plant growth and development.

**Table 3:** Functional descriptions of the sequences that Gave Rise to SSR markers associated with the BRIX, SH, SN, SW and TCH traits as determined using BLASTX and phytozome (NA: No available sequence).

**SSR marker**	**Traits**	**Description**	**e-value**
ESTA61	BRIX, SN	Cortical cell-delineating protein [*Sorghum bicolor*]	6.3E-166
ESTB111	SN	Exonuclease DPD1, chloroplastic/mitochondrial [*Sorghum bicolor*]	3.1E-113
ESTB130	SN, SW	Auxin response factor 5 [*Sorghum bicolor*]	7.0E-87
ESTB133	BRIX	Vacuolar fusion protein MON1 [*Sorghum bicolor*]	4.9E-57
ESTC19	SH	DVL family protein [*Zea mays*]	6.3E-77
CIR51	BRIX, SN, SW, TCH	Near to Cytochrome P450 transcript region [*Sorghum bicolor*]	9.1E-57
CIR55	BRIX, SN	Uncharacterized protein [*Sorghum bicolor*]	9.8E-60
SMC222	SW, TCH	NA	-
SMC248	SH	NA	-
SMC319	SH, SN, SW, TCH	NA	-

## Discussion

The complexity of the sugarcane genome and the quantitative nature of sugar- and yield-related traits are challenging for geneticists and breeders searching for higher genetic gains for this crop. Moreover, assessing genetic variables free of environmental effects and estimating their real genotypic value are extremely important for breeding purposes. Here, the genetic information obtained with SSR markers was able to efficiently distinguish ancestral and improved accessions of the BSPG due the high polymorphism and presence of unique alleles in some accessions, such as IN84-58 (*S*. *spontaneum*), Badila (*S*. *officinarum*) and Ganda Cheni (*S*. *barberi*). The identification of new alleles controlling sugar and yield metabolism in alternative *Saccharum* species and the introduction of these alleles into core germplasms would be one way to overcome obstacles in sugarcane breeding, increasing the productivity of commercial cultivars [79]. Following this strategy, association mapping is a powerful tool to identify genes and favorable alleles that could be used for the introgression process. In the present study, using the GWAS approach, we were able to detect MTAs for all five evaluated traits (BRIX, SH, SN, SW and TCH), mainly due to the presence of LD in the BSPG and by the analysis strategies employed.

The model selection approach used in this study for phenotypic data analysis can capture the heterogeneity of variance and more complex covariance structures (AR1(het)) at the genetic level, thereby improving the predictive accuracy directly related to heritability and genetic gain [12,53,80,81]. In the AR1(het) model selected for all traits (S2 Table), the correlations between harvest decay with time and each harvest have their own genetic variance [53]. Indeed, sugarcane production decreases with harvests; therefore, the differential expression of genes across harvests can be suggested. On the other hand, the use of more locations and harvest years would probably permit the adjustment of other variance and covariance structures [12].

The phenotypic range for each trait reflected the high genetic variability of the BSPG, and the broad-sense heritability values showed that much of the observed phenotypic variation can be attributed to differences at the genotypic level (Table 1). Therefore, the significant genotypic correlations among traits could indicate biological processes that are of considerable evolutionary interest and result from genetic or physiological features [82,83]. The SH, SN and SW traits are involved in plant development and are therefore important parameters in breeding programs that increase genetic gains in terms of cane yield. The MTAs discovered for any of these three traits might potentiate plant development, mainly because the SW, SH and SN traits were significantly associated with the five evaluated traits, and SW was part of the two most strongly detected correlations (SH–SW and SW–TCH) (Fig 1). Similar genotypic correlation results among these traits have been reported in previous studies [39,84].

In addition to genotypic correlations, genetic variability is essential to breeders for the generation of improved cultivars. In the present study, population structure and genetic diversity were assessed in the BSPG through DAPC analysis and by a genetic dissimilarity matrix calculated with the SM distance and visualized as an NJ phylogram, both based on SSR markers. DAPC analysis divided the BSPG into four subpopulations (Fig 2), and this result was confirmed by the NJ phylogram of the SM distance of the whole population (Fig 3). To obtain the population structure, some studies have reported similar or better results for DAPC analysis than for the Bayesian model-based method [63,85–87] implemented in STRUCTURE software [88–90]. In addition, for complex genomes, several assumptions are not fulfilled with the use of STRUCTURE; therefore, the applicability of this algorithm may be limited in sugarcane [34,37,38,91]. The NJ phylogram showed that the subpopulations contained some clusters formed by family relatedness. These results suggested that the BSPG could be affected by population structure and relatedness, which is in agreement with the history of sugarcane breeding [10,18,92].

LD is affected by genetic and nongenetic factors, such as recombination, genetic drift, population stratification, genetic relatedness, mutation, selection and linkage [93,94]. Therefore, the population structure and family relatedness of the BSPG could be responsible for the detected LD, which was stronger in the first 15 cM and present in a large extension, i.e., at 65 cM, similar to the results of Raboin et al. [35] and Wei et al. [38]. Recently, Yang et al. [36] showed a large extent of LD, with lengths of 962.4 Kbp, 2739.2 Kbp and 3573.6 Kbp for *S*. *spontaneum*, *S*. *officinarum* and modern hybrids, respectively. The existence of a large LD extent and, consequently, the presence of large gene clusters indicate that a high density of markers is not required to detect MTAs by the GWAS approach in sugarcane. Thus, single-dose markers could be useful for this purpose as an initial step. On the other hand, the LD caused by population structure and familial relatedness can promote false positive detection in GWAS analysis [95–97], and to avoid these spurious associations, the models consider covariates (population structure matrix and/or kinship matrix) to adjust the association tests on markers. In addition, confounding between these covariates and testing markers also produces false negatives [44,96].

The QQ plots obtained with GAPIT software showed that the association tests were inflated and resulted in false positives when compared with the QQ plots generated by FarmCPU software, mainly for the BRIX and SN traits (Fig 4). The compressed mixed linear model (CMLM), implemented in GAPIT, is a single-locus model that tests one marker at a time and maintains the kinship matrix constant for all markers [76]. On the other hand, FarmCPU, a multilocus model, implements a fixed model that contains the testing markers and covariates (multiple associated markers and PCs) and a random model that contains the kinship matrix. This kinship matrix is adjusted based on the testing markers and covariates of the fixed model [44]. Therefore, the differences in the analysis procedures could explain the occurrence of false positives by GAPIT, which fails to match the true genetic model of complex traits that are controlled by numerous loci simultaneously [48], such as those evaluated in the present study. In GAPIT, other associated loci nearby or elsewhere in the genome will sometimes disrupt with the tested marker and result in spurious associations, especially when the effects of the other loci are large [98]. In addition, in GAPIT, covariate information could overlap (kinship matrix and PCs), as previous studies have shown that the PCs from PCA also include part of the family relatedness [99,100]; therefore, the seven PCs retained by the DAPC analysis, which explained 25.5% of the variance, provided some information about relatedness and population structure for GWAS analysis. Finally, the more reliable MTAs detected with the FarmCPU approach could be attributed to the use of only retained PCs of DAPC as a covariate and the MLMM, which was able to remove the confounding between the tested markers and covariates [44].

The GWAS analysis with FarmCPU software revealed 23 MTAs associated with five traits when the Bonferroni-corrected threshold was set to 1% (Table 2). All but four MTAs showed a low percentage of explained phenotypic variation, with values ranging from 1% to 7%. These low values may be due to the high ploidy level of sugarcane and the quantitative inheritance of the evaluated traits [39]. In addition, the SSR fragments are treated as dominant in polyploid species, such as sugarcane, and thus do not capture the allelic dosage information of homologous chromosomes [101]. Despite that Fickett et al. [42] obtained 6299 SNPs and 235 InDels through a high-throughput genotyping system, only 27 markers were significantly associated with six traits (stalk number, stalk height, stalk diameter, °Brix, pol and fiber) and explained no more than 14.3% of the phenotypic variation. Therefore, genetic studies on polyploidy species, like sugarcane, are obviously delayed when compared to those on crops with minor genetic complexity. New methods of analysis are still in development to increase the understanding of complex genomes and enable mapping and association studies with further levels of allelic information [24,26,102]. Despite this, four MTAs with the highest percentages of explained phenotypic variation (43% for SN with ESTA61_15, 20% for BRIX with ESTA61_15, 14% for BRIX with CIR55_14 and 14% for SH with SMC248_08) indicate that the presence of at least one copy of the allele could also be important for driving strategies in breeding programs. The SSR fragment ESTA61_15, a species-specific fragment present in *S*. *spontaneum* accession IN84-58, was positively and negatively associated with the SN and BRIX traits, respectively. ESTA61_15 may be a unique allele that causes important phenotypic variation. Previous studies detected MTAs for the SW [37,39], SN [18,39,40,42], SH [18,39,40,42] and BRIX [18,40–42] traits, and the percentages of phenotypic variation found in the present study were similar for SW, SH and BRIX and higher for SN. Therefore, these MTAs may be validated as an initial approach to support breeding programs with introgression or selection processes [37,41,42].

To understand the plant metabolism functions of the SSR marker regions associated with traits and search for candidate genes, we annotated the available sequences from which the associated markers originated. Thus, the sequence that produced the ESTA61 marker showed similarity with *cortical cell-delineating protein*, which is a member of the alpha-amylase inhibitors, lipid transfer and seed storage (AAI-LTSS) protein family according to SMART annotation in Phytozome [78]. This result suggests differential lipid transport and sucrose accumulation performances between *S*. *spontaneum* and other BPSG accessions [103,104]. The ESTB133 marker, also associated with BRIX, showed similarity with the *vacuolar fusion protein MON1*, which is a member of the MON1/SAND protein family. In *Arabidopsis*, the MON1 and CCZ1 proteins form a complex that is critical for vacuolar trafficking, vacuole biogenesis, and plant growth. The *mon1* mutants show pleiotropic growth defects, fragmented vacuoles, and altered vacuolar trafficking [105]. Therefore, the accumulation and vacuolar trafficking of the sucrose in sugarcane could be affected by alteration of this marker region.

For the ESTB111 marker, which was associated with SN, similarity with *exonuclease DPD1*, *chloroplastic/mitochondrial* could indicate that the nucleotides, i.e., purines and pyrimidines, released during the leaf senescence process provide nitrogen, sugar and phosphate to maintain or increase the plant tillering ability [106]. Likewise, the ESTB130 marker, which was associated with SN and SW, showed similarity to *auxin response factor 5* (ARF5), which acts as a transcriptional activator of auxin-responsive promoter elements. This homology suggests that a modification in the ARF5 protein could affect plant growth and development and consequently affect the weight and stalk production of sugarcane [107–109]. For the SH trait, the significantly associated marker ESTC19 showed similarity to DVL family proteins. In *Arabidopsis*, the overexpression of DVL1 was associated with plants with a shortened stature, smaller and rounder rosette leaves, clustered inflorescences, shortened pedicles, and siliques with pronged tips resembling horns [110]. Thus, this result suggests that the ESTC19 marker also plays a role in sugarcane plant development.

The GWAS analysis with FarmCPU software, which used population structure information derived from DAPC analysis as a covariate, was able to detect MTAs with efficient control of spurious associations in sugarcane. In addition, the verification of possible candidate genes for MTAs showed the importance of providing insights into gene networks that are related to the expression of target traits. This approach has great potential for assisting breeding programs in increasing the genetic gain rate of target traits. However, the development of statistical approaches to enable mapping association with markers in multiple doses is important to enhance the probability of finding higher numbers of significant associations and, consequently, increase the use of molecular markers in breeding programs of outcrossing heterozygous species, such as sugarcane.

## Supporting information

S1 Table Names, parents and origins of 134 accessions of the BPSG. (PDF)

S2 Table Selected models for the G_*M*_ matrix and number of estimated parameters (*n*_*par*_) considering each trait separately. The Akaike (AIC) and Bayesian (BIC) information criteria were used to compare the structures of the variance–covariance matrix. The models for the **G**_*M*_ matrix were selected according to the lowest value of the BIC criterion for BRIX as °Brix, stalk height (SH) in m, stalk number (SN) by direct counting, stalk weight (SW) in kg and cane yield (TCH) in t ha-1 for BPSG over two harvest years (plant cane and first ratoon). Bold numbers represent the smallest AIC and BIC values.
(PDF)

S3 Table SSR fragments with the largest values of contribution to subpopulation identification detected through the loadingplot function. A threshold of 0.005 was used to declare the major contributions. LD: linear discriminant.
(PDF)

S1 Fig Number of subpopulations (clusters) vs. BIC values. The x-axis represents the different number of subpopulations that could be presented in the Brazilian Panel of Sugarcane Genotypes (BPSG). The y-axis represents the BIC value associated with each number of subpopulations.
(PDF)

S2 Fig Cross validation of DAPC. The x-axis represents the number of PCs retained in each DAPC. The y-axis represents the proportion of successful outcome prediction. Each dot represents the individual replicate of the analysis.
(PDF)

S3 Fig Numbers of retained principal components (PCs) vs. α-score values. The α-score on the y-axis depicts the difference between the proportion of successful reassignment of the analysis (observed discrimination) and the values obtained using random groups (random discrimination). The x-axis represents the number of retained PCs for each random group. The spline interpolation approximates the optimal number of PCs to be retained.
(PDF)

S4 Fig Distribution of the accessions of BPSG (y-axis) into 4 subpopulations (x-axis) obtained through the DAPC. The red regions inside the columns indicate the set of accessions grouped in the corresponding subpopulation according to the membership probabilities.
(PDF)

S5 Fig Variable contributions (SSR Fragments) for linear discriminants 1 (LD1) and 2 (LD2). A threshold of 0.005 was used to declare major contributions.
(PDF)

S6 Fig Heatmap with genetic dissimilarities among accessions of the BPSG. The subpopulations obtained through discriminant analysis of principal components (DAPC) are also shown.
(PDF)

S7 Fig Plot of linkage disequilibrium (−*LogP*) and genetic distance (cM) in the SP80-180 and SP80-4966 genetic map. The thresholds corresponding to the Bonferroni and false discovery rate (FDR) corrections are indicated on a logarithmic scale, showing significant and nonsignificant associations above and below their values, respectively. Genetic distances were obtained through the Kosambi mapping function.
(PDF)
