# Supplementary material for: A genome-wide association study identified loci for yield component traits in sugarcane (Saccharum spp.)
Source: PLoS One. 2019 Jul 18;14(7):e0219843. doi: 10.1371/journal.pone.0219843 (PMC6638961; doi:10.1371/journal.pone.0219843)
Supplement: S1 Table — (PDF) [file pone.0219843.s001.pdf]

S1 Table. Names, Parents and Origins of 134 Accessions of the BPSG.

Accessions	Parent 1	Parent 2	Origin
EK28	EK2	POJ100	Java
Badila	*S. officinarum*	NG9615	New Guinea
CB36-24	POJ2878	?	Brazil
CB40-13	POJ2878	Co290	Brazil
CB41-76	POJ2878	?	Brazil
CB45-155	Co413	?	Brazil
CB45-3	Co331	Co290	Brazil
CB46-47	POJ2878	?	Brazil
CB47-355	POJ2878	Co413	Brazil
CB49-260	CB44-36	?	Brazil
CB53-98	CB46-40	?	Brazil
Co290	Co221	D74	India
Co331	Co213	Co214	India
Co419	POJ2878	Co290	India
Co449	POJ2878	Co331	India
Co740	P3247	P4775	India
Co997	Co683	P63-32	India
CP51-22	F36-819	CP33-372	USA
CP52-68	CP29-320	CP38-34	USA
CP53-76	F36-819	CP36-46	USA
CP70-1547	CP62-374	CP57-526	USA
F31-962	Co-281	CP27-108	Taiwan
F36-819	F31-962	POJ2878	Taiwan
Ganda Cheni	*S. barberi*	*S. barberi*	India
H53-3989	H48-3717	?	Hawaii
H59-1966	H50-676	H49-3646	Hawaii
IAC48-65	CP27-108	?	Brazil
IAC49-131	CP27-108	?	Brazil
IAC50-134	Co419	Co285	Brazil
IAC51-205	POJ2878	?	Brazil
IAC52-150	Co419	Co285	Brazil
IAC58-480	POJ2878	CP44-101	Brazil
IAC64-257	Co419	IAC49-131	Brazil
IAC68-12	Co419	IAC52-179	Brazil
IAC82-2045	IAC65-113	IAC52-150	Brazil
IAC82-3092	CB41-76	IAC68-12	Brazil
IAC83-4157	IAC68-12	SP70-1143	Brazil
IAC86-2210	CP52-48	Co798	Brazil
IAC87-3396	Co740	SP70-1143	Brazil
IAC91-1099	RB785148	?	Brazil
IN84-58	*S. spontaneum*		
L60-14	CP52-1	CP48-103	USA
Maneria	*S. sinense*	*S. sinense*	
NA56-79	Co419	SELF	Argentina
NCo310	Co421	Co312	India
POJ2878	POJ2364	EK28	Indonesia
R570	H32-8560	R445	Reunion
RB721012	Co331	?	Brazil
RB72199	NCo334	?	Brazil
RB72454	CP53-76	?	Brazil
RB725053	Co775	?	Brazil
RB725828	NA56-79	?	Brazil
RB732577	NCo376	?	Brazil
RB735200	Co331	?	Brazil
RB735220	CB44-95	?	Brazil

S1 Table. Continued.

Accessions	Parent 1	Parent 2	Origin
RB735275	IAC49-131	?	Brazil
RB739359	IANE55-34	?	Brazil
RB739735	CB52-179	?	Brazil
RB75126	C278	?	Brazil
RB765418	M253/48	?	Brazil
RB785148	IAC47-31	?	Brazil
RB806043	NA56-79	?	Brazil
RB815690	IAC49-131	NA56-79	Brazil
RB825317	L60-14	CB47-355	Brazil
RB825336	H53-3989	?	Brazil
RB83102	NA56-79	SP70-1143	Brazil
RB835019	RB72454	NA56-79	Brazil
RB835054	RB72454	NA56-79	Brazil
RB835089	RB72454	NA56-79	Brazil
RB835205	Co740	?	Brazil
RB835486	L60-14	?	Brazil
RB845197	RB72454	SP70-1143	Brazil
RB845210	RB72454	SP70-1143	Brazil
RB845257	RB72454	SP70-1143	Brazil
RB855002	SP70-1143	RB72454	Brazil
RB855035	L60-14	SP70-1284	Brazil
RB855036	RB72454	SP70-1143	Brazil
RB855077	SP70-1143	TUC71-7	Brazil
RB855113	SP70-1143	RB72454	Brazil
RB855156	RB72454	TUC71-7	Brazil
RB855206	RB72454	TUC71-7	Brazil
RB855350	RB72454	?	Brazil
RB855453	TUC71-7	?	Brazil
RB855463	RB72454	?	Brazil
RB855465	RB72454	?	Brazil
RB855511	SP71-1406	?	Brazil
RB855536	SP70-1143	RB72454	Brazil
RB855546	SP70-1143	RB72454	Brazil
RB855563	TUC71-7	SP70-1143	Brazil
RB855589	SP70-1143	TUC71-7	Brazil
RB855595	SP70-1143	TUC71-7	Brazil
RB867515	RB72454	?	Brazil
RB925211	RB855206	?	Brazil
RB925268	RB855511	?	Brazil
RB925345	H59-1966	?	Brazil
RB92579	RB75126	RB72199	Brazil
RB935744	RB835089	RB765418	Brazil
RB965902	RB855536	RB855453	Brazil
RB965917	RB855453	RB855453	Brazil
RB966928	RB855156	RB815690	Brazil
SP70-1005	IAC48-65	?	Brazil
SP70-1078	IAC48-65	?	Brazil
SP70-1143	IAC48-65	?	Brazil
SP70-1284	CB41-76	?	Brazil
SP70-1423	CB41-76	?	Brazil
SP70-3370	CP53-17	?	Brazil
SP71-1406	NA56-79	?	Brazil
SP71-6163	NA56-79	?	Brazil
SP71-6949	NA56-79	?	Brazil
SP71-799	NA56-79	?	Brazil
SP72-4928	CP52-48	?	Brazil
SP77-5181	HJ57-41	?	Brazil
SP79-1011	NA56-79	Co775	Brazil

S1 Table. Continued.

Accessions	Parent 1	Parent 2	Origin
SP79-2233	H56-2954	?	Brazil
SP79-2312	SP71-6106	?	Brazil
SP79-2313	SP71-6106	?	Brazil
SP79-6134	H63-4644	?	Brazil
SP79-6192	SP70-3518	?	Brazil
SP80-1520	H48-3166	SP71-1088	Brazil
SP80-180	B3337	?	Brazil
SP80-1816	SP71-1088	H57-5028	Brazil
SP80-1836	SP71-1088	H57-5028	Brazil
SP80-1842	SP71-1088	H57-5028	Brazil
SP80-185	BO17	IAC50-134	Brazil
SP80-3280	SP71-1088	H57-5028	Brazil
SP80-4966	SP71-1406	?	Brazil
SP81-1763	Co775	NA56-79	Brazil
SP81-3250	CP70-1547	SP71-1279	Brazil
SP83-2847	HJ57-41	SP70-1143	Brazil
SP83-5073	SP71-1406	SP71-1088	Brazil
SP89-1115	CP73-1547	?	Brazil
SP91-1049	SP80-3328	SP81-3250	Brazil
TUC71-7	CP52-68	CP62-258	Argentina
White Transparent	*S. officinarum* spp		India
