# Supplementary material for: A genome-wide association study identified loci for yield component traits in sugarcane (Saccharum spp.)
Source: PLoS One. 2019 Jul 18;14(7):e0219843. doi: 10.1371/journal.pone.0219843 (PMC6638961; doi:10.1371/journal.pone.0219843)
Supplement: S2 Table — The Akaike (AIC) and Bayesian (BIC) information criteria were used to compare the structures of the variance–covariance matrix. The models for the GM matrix were selected according to the lowest value of the BIC criterion for BRIX as °Brix, stalk height (SH) in m, stalk number (SN) by direct counting, stalk weight (SW) in kg and cane yield (TCH) in t ha-1 for BPSG over two harvest years (plant cane and first ratoon). Bold numbers represent the smallest AIC and BIC values. (PDF) [file pone.0219843.s002.pdf]

S2 Table. Selected Models for the $\mathbf{G}_M$ Matrix and Number of Estimated Parameters (n_{par}) Considering Each Trait Separately. The Akaike (AIC) and Bayesian (BIC) information criteria were used to compare the structures of the variance–covariance matrix. The models for the $\mathbf{G}_M$ matrix were selected according to the lowest value of the BIC criterion for BRIX as °Brix, stalk height (SH) in m, stalk number (SN) by direct counting, stalk weight (SW) in kg and cane yield (TCH) in t ha-1 for BPSG over two harvest years (plant cane and first ratoon). Bold numbers represent the smallest AIC and BIC values.

Trait	$\mathbf{G}_M$ matrix	Models	n_{par}	AIC	BIC
BRIX (°Brix)	$\mathbf{G}_M = \mathbf{G}_{M\,x\,M}$	(1) ID	1	3191.28	3215.75
		(2) DIAG	2	3189.78	3219.13
		(3) AR1	2	*	*
		(4) AR1(het)	2	**3103.14**	**3137.38**
SH (m)	$\mathbf{G}_M = \mathbf{G}_{M\,x\,M}$	(1) ID	1	500.28	524.77
		(2) DIAG	2	502.51	531.51
		(3) AR1	2	*	*
		(4) AR1(het)	2	**359.93**	**394.22**
SN	$\mathbf{G}_M = \mathbf{G}_{M\,x\,M}$	(1) ID	1	9268.75	9293.21
		(2) DIAG	2	9252.54	9281.89
		(3) AR1	2	*	*
		(4) AR1(het)	2	**9089.19**	**9123.44**
SW (Kg)	$\mathbf{G}_M = \mathbf{G}_{M\,x\,M}$	(1) ID	1	10515.65	10540.15
		(2) DIAG	2	10514.88	10544.28
		(3) AR1	2	*	*
		(4) AR1(het)	2	**10378.72**	**10413.01**
TCH (t ha^{-1})	$\mathbf{G}_M = \mathbf{G}_{M\,x\,M}$	(1) ID	1	*	*
		(2) DIAG	2	*	*
		(3) AR1	2	*	*
		(4) AR1(het)	2	**11071.67**	**11106.08**
