# Supplementary material for: A genome-wide association study identified loci for yield component traits in sugarcane (Saccharum spp.)
Source: PLoS One. 2019 Jul 18;14(7):e0219843. doi: 10.1371/journal.pone.0219843 (PMC6638961; doi:10.1371/journal.pone.0219843)
Supplement: S3 Table — A threshold of 0.005 was used to declare the major contributions. LD: linear discriminant. (PDF) [file pone.0219843.s003.pdf]

S3 Table. SSR Fragments with the Largest Values of Contribution to Subpopulation Identification Detected Through the Loadingplot Function. A threshold of 0.005 was used to declare the major contributions. LD: linear discriminant.

LD	Code	Marker fragment	Values
LD1	M45	CIR21_24	0.00549
LD1	M46	CIR21_25	0.00575
LD1	M143	ESTB67_01	0.00583
LD1	M149	ESTB67_07	0.00566
LD1	M373	CIR18_10	0.00608
LD1	M385	CIR18_22	0.0051
LD1	M408	ESTA31_18	0.00581
LD1	M409	ESTA31_19	0.00675
LD1	M533	ESTB47_04	0.00539
LD1	M572	ESTB45_12	0.00611
LD1	M646	ESTB111_01	0.00714
LD1	M688	ESTC22_01	0.00681
LD1	M748	SMC248_04	0.00532
LD1	M956	ESTB125_10	0.00656
LD1	M959	ESTB125_13	0.00543
LD1	M960	ESTB125_14	0.0062
LD1	M1141	ESTC130_17	0.00547
LD1	M1147	ESTA70_05	0.00522
LD1	M1173	ESTC77_10	0.00577
LD1	M1190	ESTB106_01	0.0056
LD1	M1202	ESTC124_06	0.00537
LD1	M1232	ESTC81_13	0.00654
LD1	M1306	ESTC52_04	0.00586
LD1	M1357	ESTB66_07	0.00506
LD2	M20	CIR23_20	0.0091
LD2	M24	CIR21_03	0.00531
LD2	M52	ESTA26_04	0.00501
LD2	M163	ESTB68_01	0.00523
LD2	M281	ESTA49_10	0.00507
LD2	M298	ESTA33_04	0.00643
LD2	M308	ESTA94_07	0.00614
LD2	M508	ESTA54_13	0.00507
LD2	M729	ESTC60_09	0.00561
LD2	M746	SMC248_02	0.00549
LD2	M792	ESTB133_05	0.00555
LD2	M845	ESTB73_04	0.00658
LD2	M855	ESTB73_14	0.00865

LD	Code	Marker fragment	Values
LD2	M964	ESTB125_18	0.00675
LD2	M1125	ESTC130_01	0.00796
LD2	M1171	ESTC77_08	0.00741
LD2	M1339	ESTC35_02	0.00516
LD2	M1425	ESTC84_10	0.00673
