# Supplementary figures and images for: A genome-wide association study identified loci for yield component traits in sugarcane (Saccharum spp.)
Source: PLoS One. 2019 Jul 18;14(7):e0219843. doi: 10.1371/journal.pone.0219843 (PMC6638961; doi:10.1371/journal.pone.0219843)

**Value of BIC  
versus number of clusters**

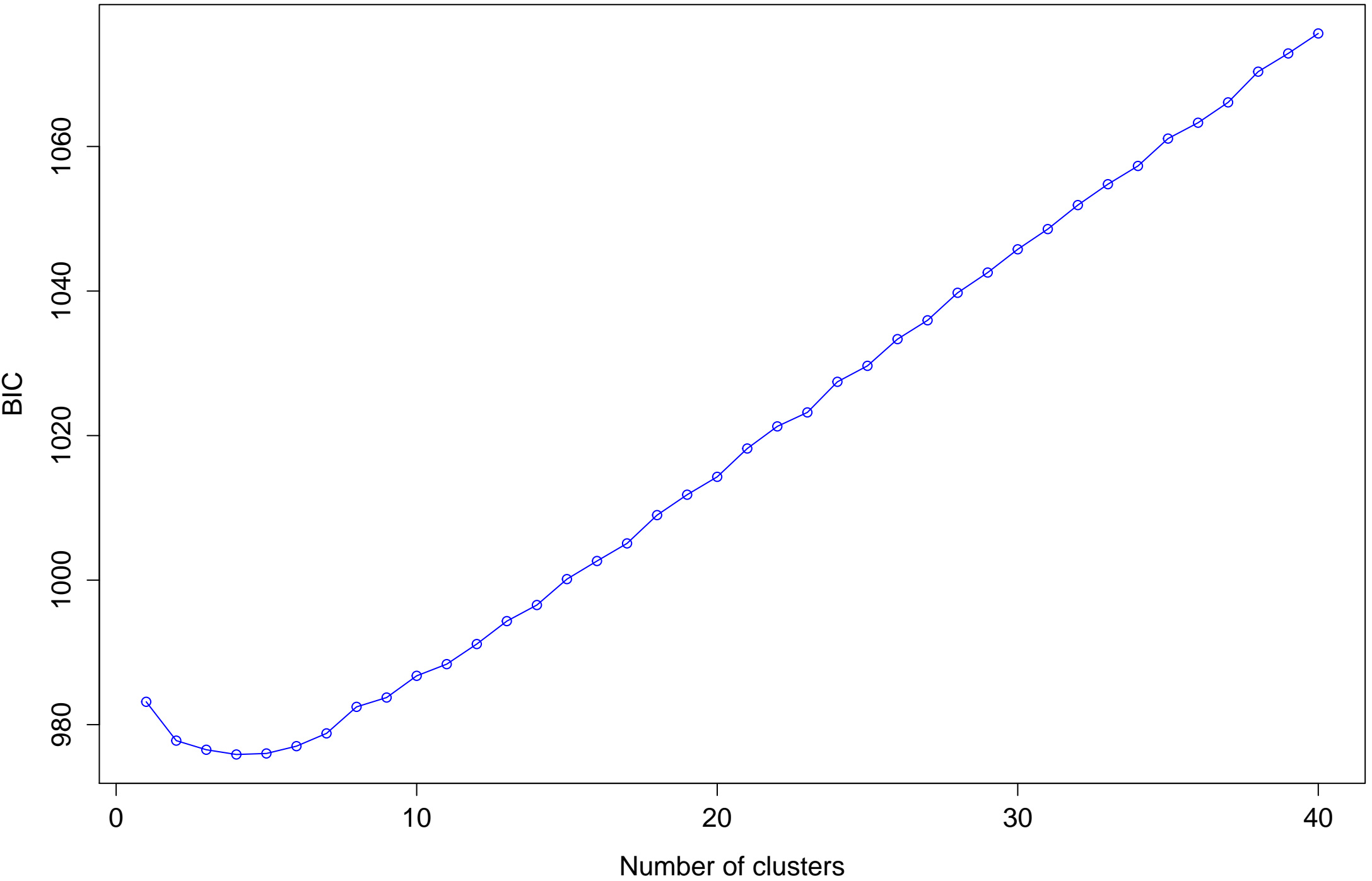

Supplement: S1 Fig — The x-axis represents the different number of subpopulations that could be presented in the Brazilian Panel of Sugarcane Genotypes (BPSG). The y-axis represents the BIC value associated with each number of subpopulations. (PDF) [file pone.0219843.s004.pdf]

## DAPC Cross-Validation

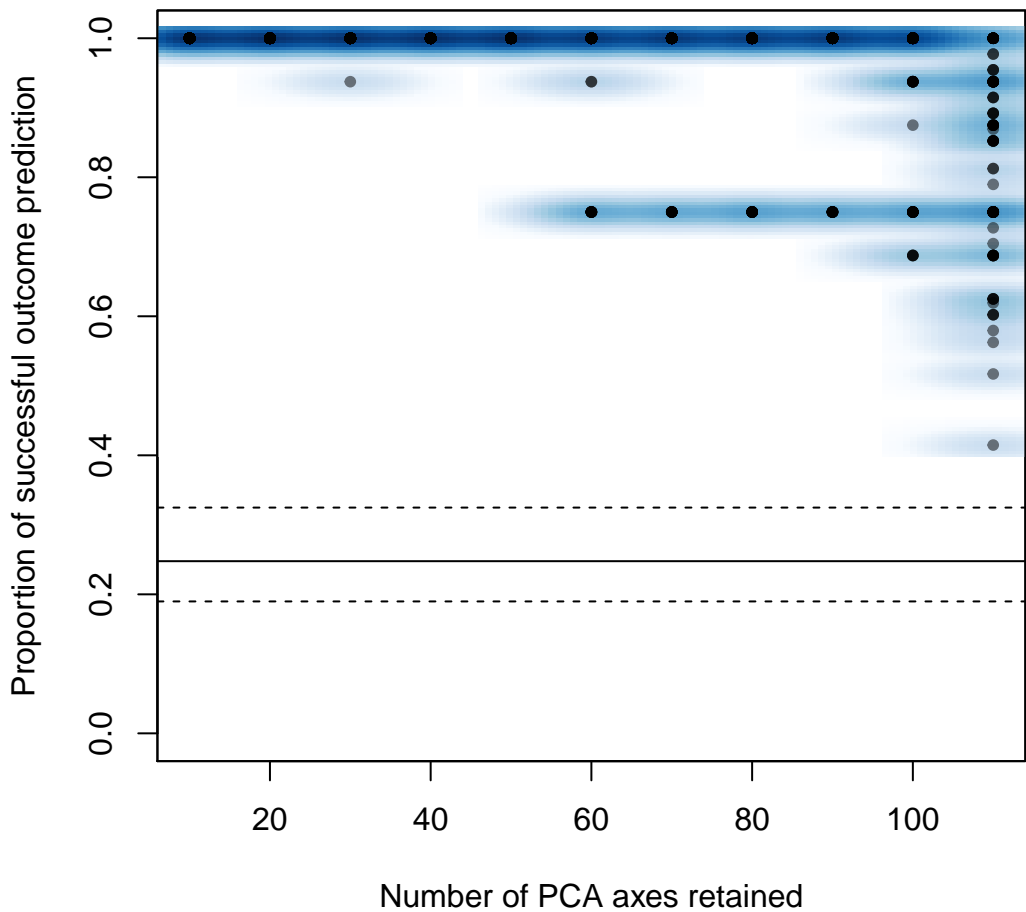

Supplement: S2 Fig — The x-axis represents the number of PCs retained in each DAPC. The y-axis represents the proportion of successful outcome prediction. Each dot represents the individual replicate of the analysis. (PDF) [file pone.0219843.s005.pdf]

# a-score optimisation – spline interpolation

Optimal number of PCs: 7

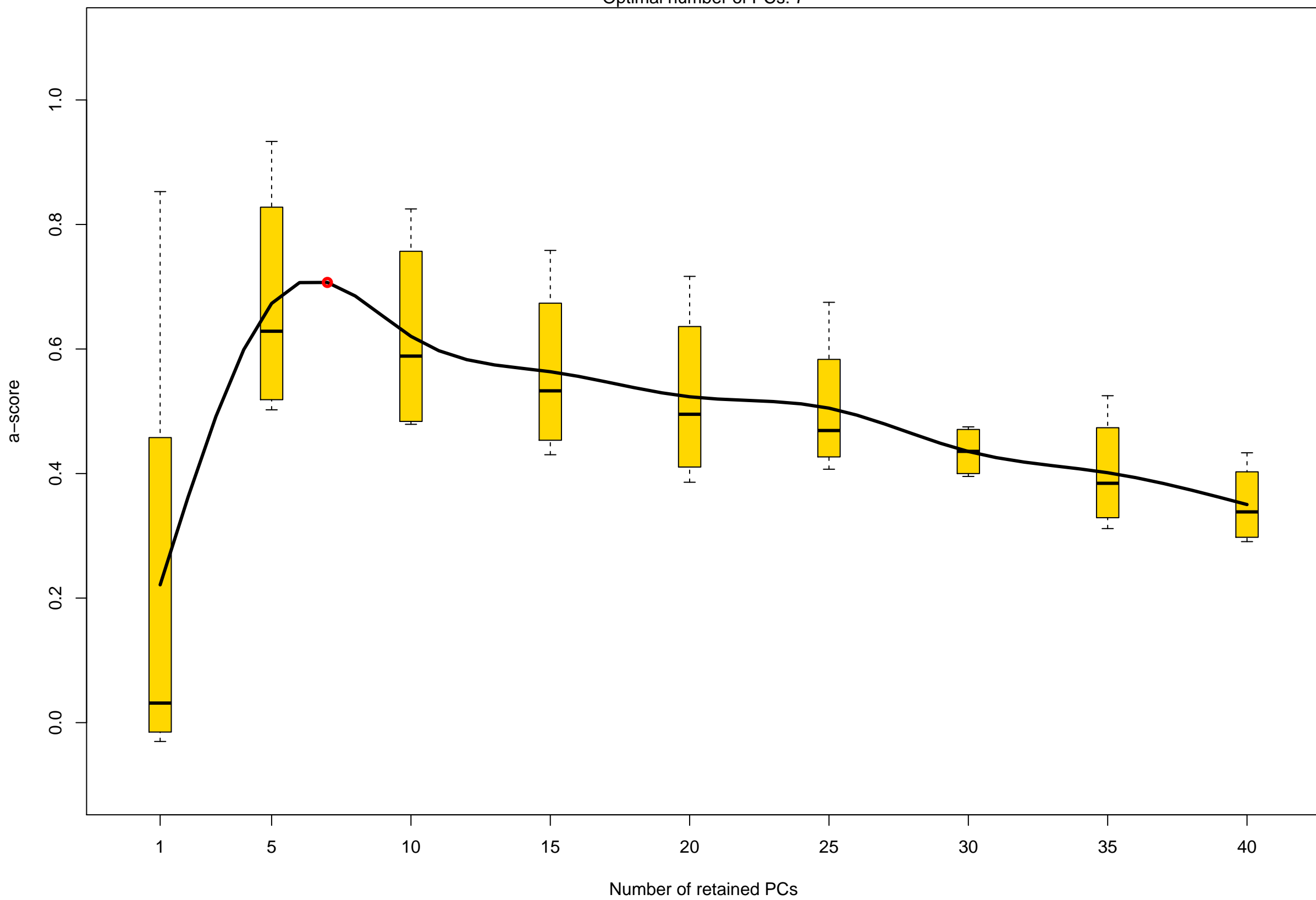

Supplement: S3 Fig — The α-score on the y-axis depicts the difference between the proportion of successful reassignment of the analysis (observed discrimination) and the values obtained using random groups (random discrimination). The x-axis represents the number of retained PCs for each random group. The spline interpolation approximates the optimal number of PCs to be retained. (PDF) [file pone.0219843.s006.pdf]

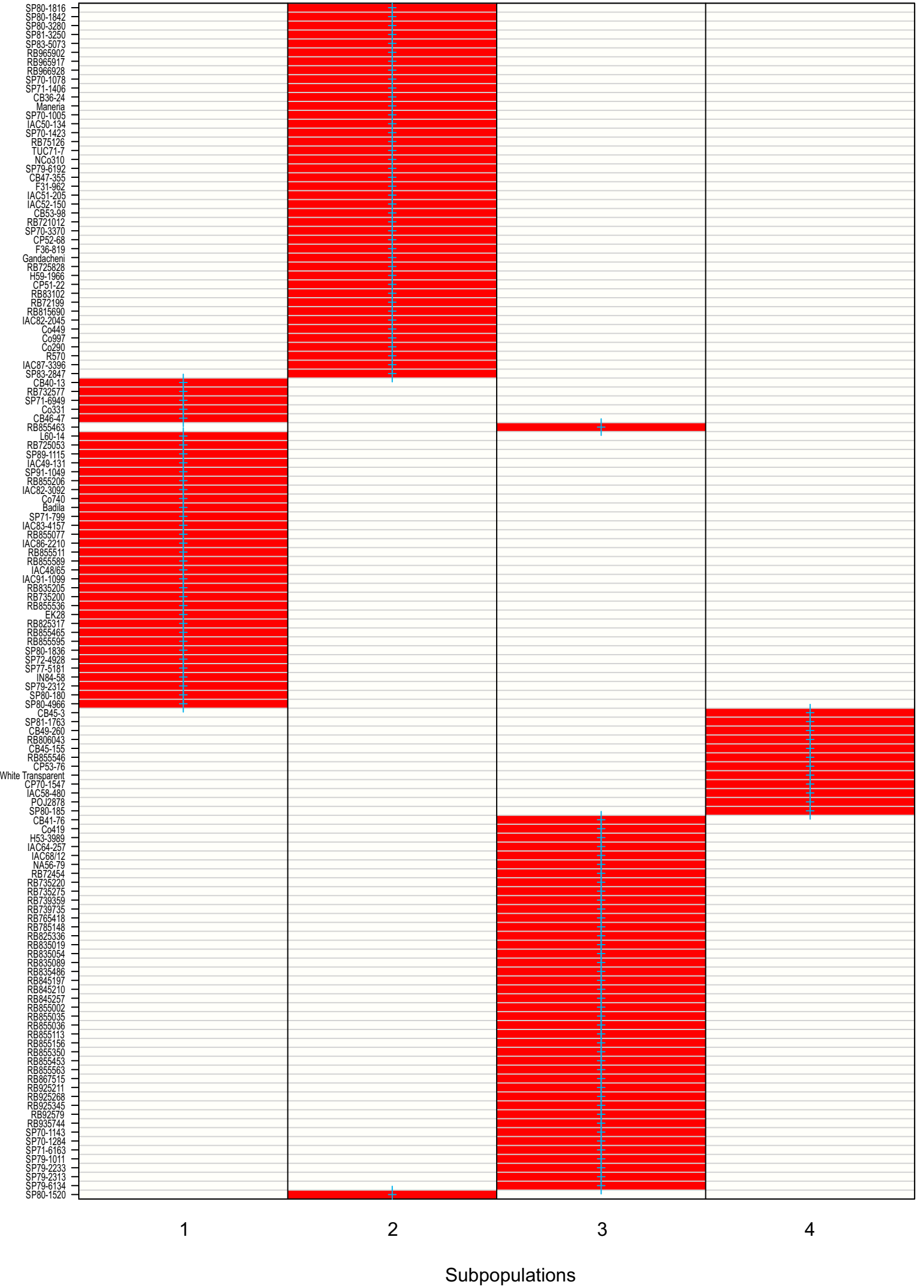

Supplement: S4 Fig — The red regions inside the columns indicate the set of accessions grouped in the corresponding subpopulation according to the membership probabilities. (PDF) [file pone.0219843.s007.pdf]

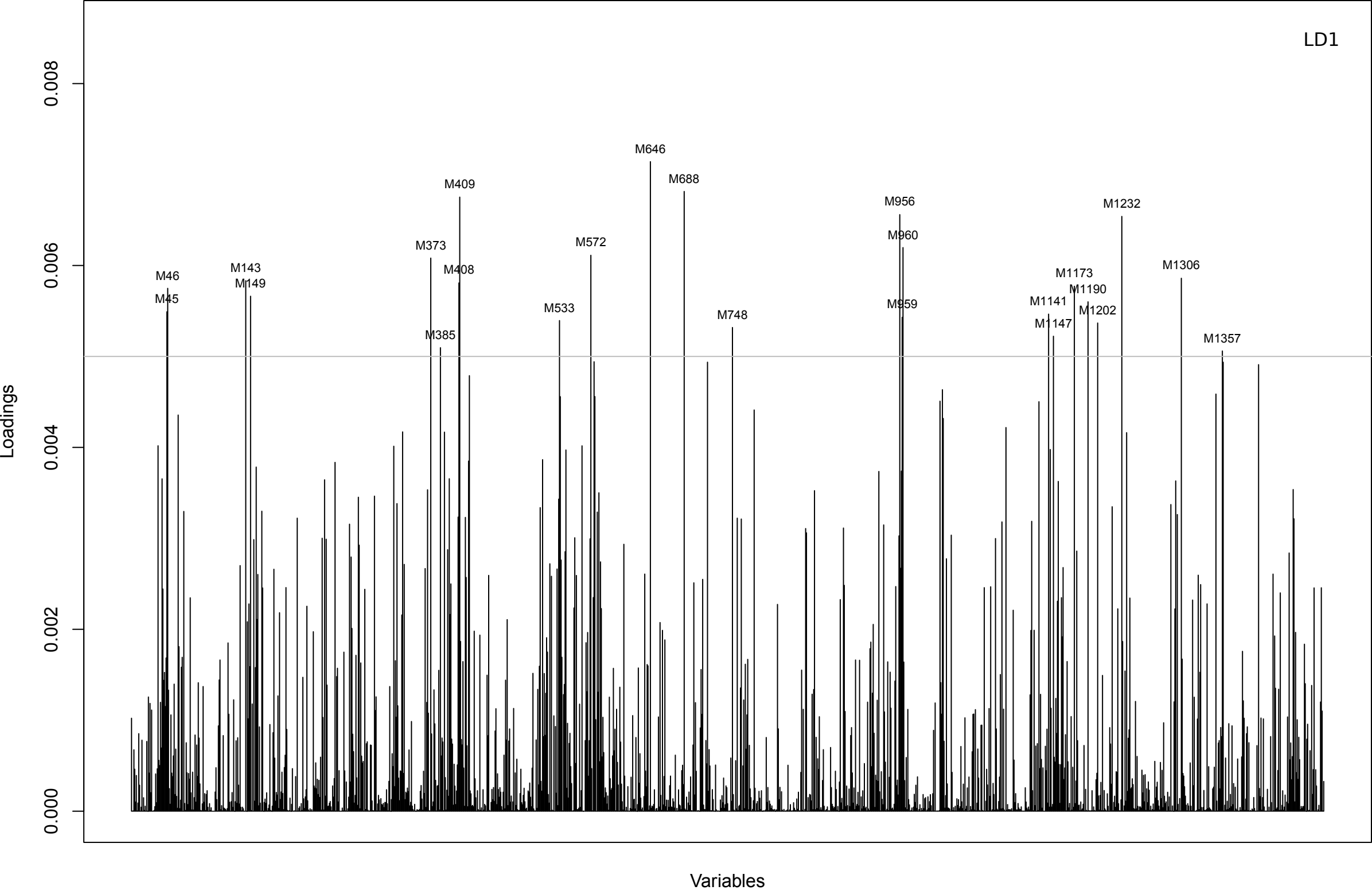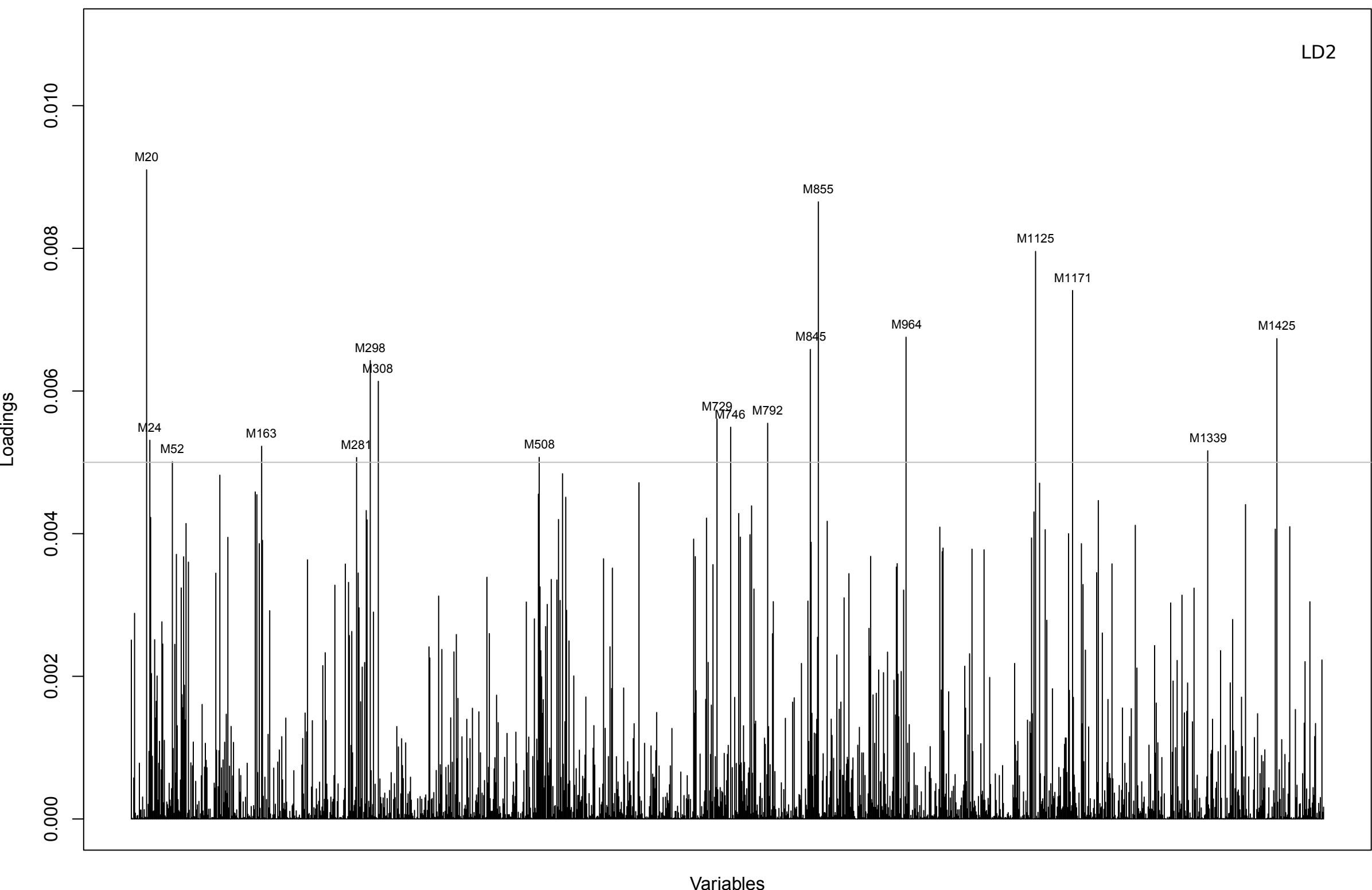

Supplement: S5 Fig — A threshold of 0.005 was used to declare major contributions. (PDF) [file pone.0219843.s008.pdf]

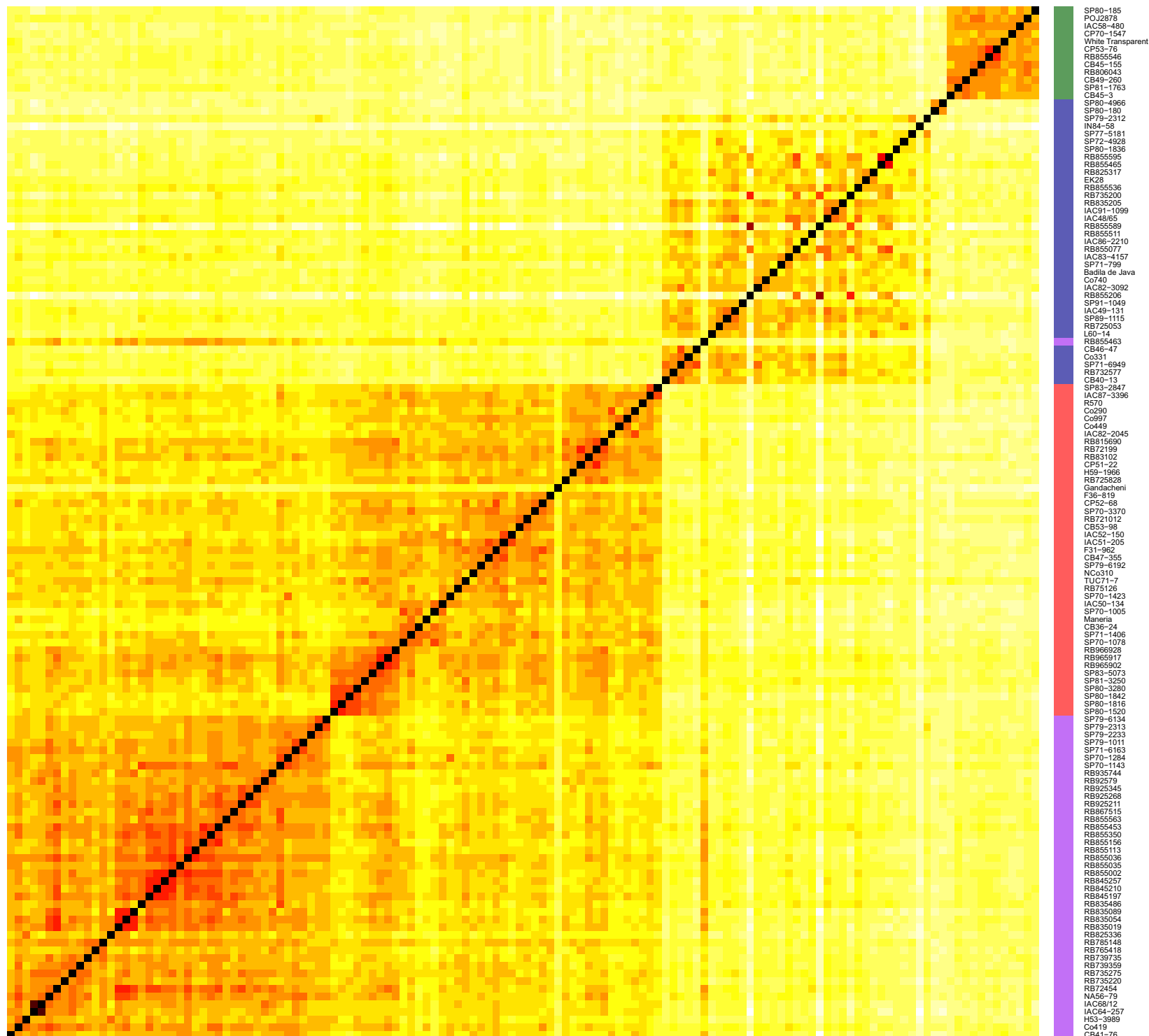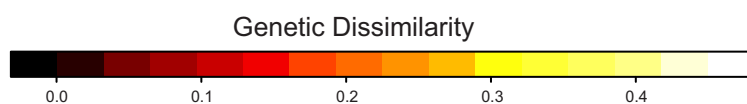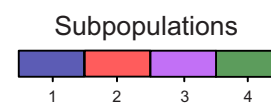

Supplement: S6 Fig — The subpopulations obtained through discriminant analysis of principal components (DAPC) are also shown. (PDF) [file pone.0219843.s009.pdf]

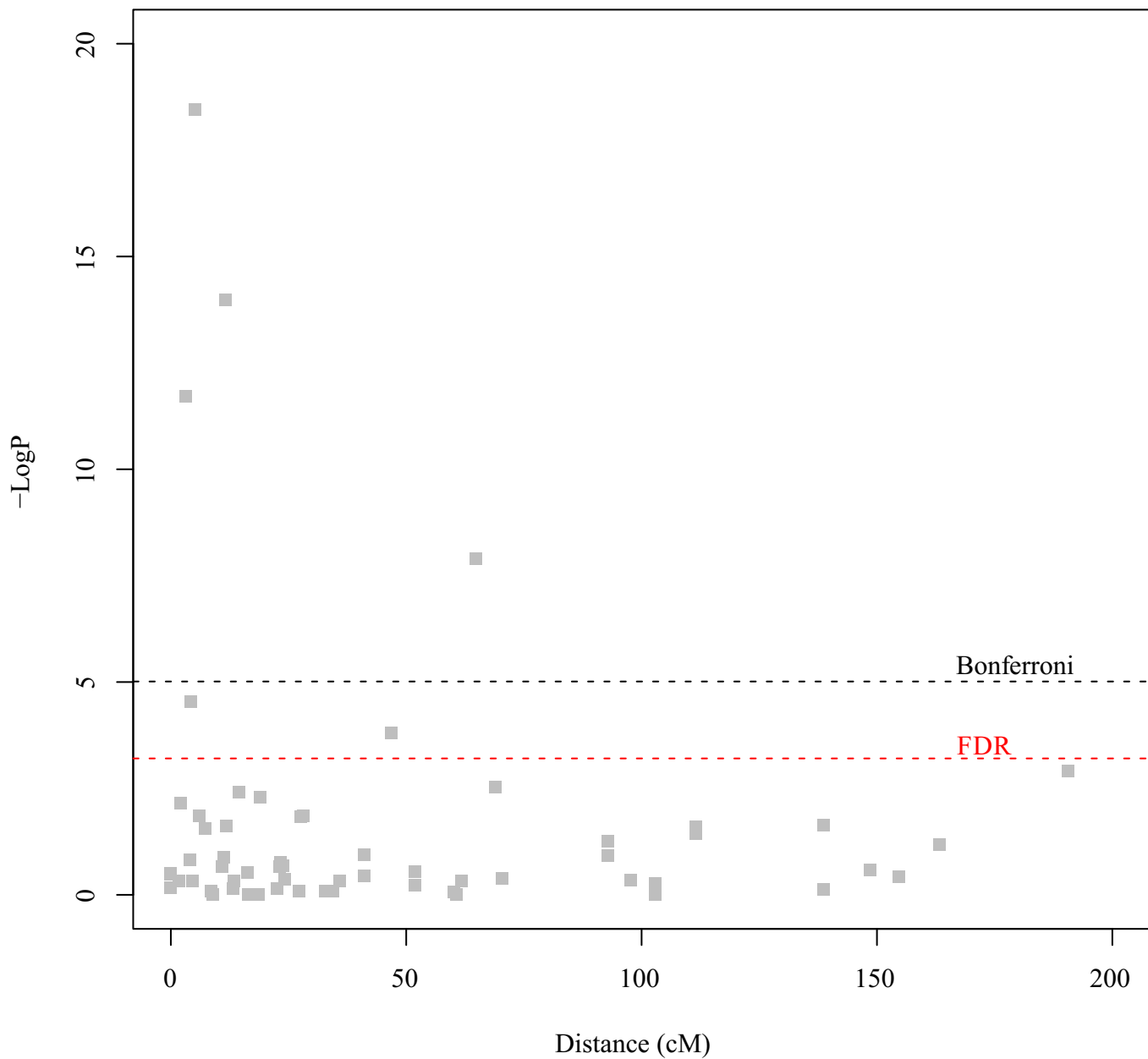

Supplement: S7 Fig — The thresholds corresponding to the Bonferroni and false discovery rate (FDR) corrections are indicated on a logarithmic scale, showing significant and nonsignificant associations above and below their values, respectively. Genetic distances were obtained through the Kosambi mapping function. (PDF) [file pone.0219843.s010.pdf]
